# Multicomponent Nutritional Approach (NutrirCom) and Its Effects on Anthropometric, Metabolic, and Psychoemotional Outcomes in Women with Obesity: A Three-Arm Randomized Clinical Trial

**DOI:** 10.3390/nu18030414

**Published:** 2026-01-27

**Authors:** Irene da Silva Araújo Gonçalves, Tatiana do Nascimento Campos, Dayse Mara de Oliveira Freitas, Leticia Paiva Milagres, Marina Tosatti Aleixo, Ana Clara Gutierrez Souza Lacerda, Tiago Ricardo Moreira, Danielle Cabrini, Bianca Guimarães de Freitas, Jéssica Aparecida da Silva, Monica de Paula Jorge, Nicolly Oliveira Custodio, Rosangela Minardi Mitre Cotta, Glauce Dias da Costa

**Affiliations:** 1 Department of Nutrition and Health, Universidade Federal de Viçosa, Viçosa 36570-900, MG, Brazilglauce.costa@ufv.br (G.D.d.C.); 2 College of Nutrition, Universidade Federal de Ouro Preto, Ouro Preto 35400-000, MG, Brazil; 3 D’Or Institute for Research and Education (IDOR), Rio de Janeiro 22281-100, RJ, Brazil; 4 Department of Medicine and Nursing, Universidade Federal de Viçosa, Viçosa 36570-900, MG, Brazil; 5 Health Sciences Center, Universidade Federal do Espírito Santo, Vitória 29075-910, ES, Brazil

**Keywords:** protocol, multicomponent approach, obesity, women, nondiet approach, weight inclusivity

## Abstract

**Background/Objectives**: Obesity is a multifactorial condition and a major public health challenge. Conventional treatment centers on caloric restriction, which is often unsustainable and may cause stigma and psychoemotional harm. This study aimed to describe the methodology and assess the effects of a multicomponent nutritional intervention not focused on caloric restriction on psychoemotional outcomes. Women were selected as the target population because of the higher prevalence of obesity-related psychoemotional distress, body dissatisfaction, and weight-related stigma in this group, as well as their greater vulnerability to the psychosocial impacts of weight-focused interventions. **Methods**: This randomised, parallel, open-label trial included 89 obese women from primary care in Viçosa, Brazil. The participants were allocated into three groups: Group 1 (Control), which received a personalised hypocaloric diet (from 500 to 1000 kcal/day); Group 2 (NutrirCom (NutrirCom is a multicomponent, person-centred nutritional intervention protocol that is not focused on caloric restriction, conceived by a group of researchers at the Federal University of Viçosa for the care of women with obesity in Primary Health Care. It integrates nutritional, psychoemotional, behavioural, and social strategies, with an emphasis on promoting eating autonomy, mental health, and quality of life through a humanised, integrated, and sustainable approach, aiming to enhance the effectiveness of health care delivery and clinical practice)), which received 10 individual NutrirCom-based sessions; and Group 3 (NutrirCom + Social Support), which combined individual NutrirCom sessions with monthly group meetings for social support. Randomisation was stratified by body mass index via Excel^®^ with concealed allocation. The six-month intervention assessed changes in stress, anxiety, depression, and self-compassion, along with anthropometric and metabolic markers. **Results**: All groups presented reductions in waist circumference, fasting glucose, and total body fat, with increased lean mass. Anxiety remained unchanged in Group 1 but decreased significantly in Groups 2 (*p* = 0.002) and 3 (*p* = 0.005). Only Group 2 showed a significant reduction in depression symptoms (*p* = 0.023). Self-compassion improved significantly in groups 2 and 3. **Conclusions**: NutrirCom is a low-cost, scalable, and human-centered intervention that integrates emotional, social, and nutritional aspects of care. This approach shows promise as a sustainable strategy for obesity treatment in primary health care. Registration: Brazilian Registry of Clinical Trials (ReBEC) (no. RBR-87wb8x5).

## 1. Introduction

Obesity is a multifactorial chronic condition and constitutes one of the most pressing global public health challenges. It is associated with an elevated risk of cardiovascular disease, type 2 diabetes, and psychological disorders and with diminished quality of life and life expectancy [1,2].

Traditionally, its clinical management prioritises weight loss through hypocaloric diets [3]. However, this approach has demonstrated limited long-term efficacy and may heighten vulnerability to eating disorders and psychological distress [4,5,6]. Dietary restriction increases appetite, a sense of deprivation, and cognitive control over eating, thereby fostering episodes of binge eating and perpetuating the cycle of dieting, loss of control, and guilt [7,8].

Currently, obesity management encompasses a diverse set of therapeutic strategies, including lifestyle interventions, pharmacological treatments, surgical procedures, and innovative models of health care delivery. Lifestyle-based interventions remain the cornerstone of treatment and typically involve nutritional counselling, the promotion of physical activity, and behavioral support. These interventions are delivered by multiprofessional teams composed of nutritionists, physicians, psychologists, nurses, and physical education professionals. More recently, telemedicine and digital health interventions have been established as complementary strategies in obesity care, expanding access to services and facilitating longitudinal follow-up, self-monitoring, and behavioral support, particularly within the context of primary health care [9]. Recent evidence indicates that remote or hybrid models can be effective when integrated into comprehensive and coordinated approaches, reinforcing the importance of multiprofessional, continuous interventions that are sensitive to the social and cultural context of individuals with obesity [10].

Importantly, calorie-restriction-based approaches occupy a central position in the scientific literature on obesity treatment and have been extensively tested across different clinical settings, demonstrating effectiveness, particularly in reducing body weight in the short and medium term [11]. These studies have contributed substantially to the understanding of the physiological and behavioral mechanisms involved in weight control and have informed current clinical practice. However, evidence accumulated over recent decades has also highlighted limitations related to long-term maintenance of outcomes, sustained adherence, and psychosocial impacts, particularly among women [12]. Recognition of these limitations does not invalidate previous findings but rather underscores gaps that motivated the development of the present study, which seeks to expand care by incorporating emotional, behavioral, and contextual dimensions through a multicomponent nutritional approach.

Moreover, the societal valorisation of thinness contributes to body stigma and dissatisfaction with body image, particularly among women [13]. Women living with obesity encounter additional barriers to care, involving hormonal, metabolic, psychosocial, and contextual factors that compromise adherence and reduce the effectiveness of conventional interventions [14].

Strategies focused exclusively on weight loss do not yield sustainable benefits and may adversely affect psychological well-being [15,16,17,18]. In this context, multicomponent nutritional interventions have emerged as promising alternatives by integrating educational initiatives, emotional support, the promotion of physical activity, and social engagement. These approaches prioritise health and quality of life without centring on weight loss [19,20]. Their components include the encouragement of healthy food choices, body acceptance, tailored physical activity, and social inclusion [21,22].

To increase the effectiveness of these interventions, it is essential to recognise the social determinants of health as structural elements of care [23].

Multicomponent nutritional interventions are grounded in behavioral theories such as Social Cognitive Theory, which conceptualizes health behavior as the interaction of personal, environmental, and behavioral factors [24], and ecological models that emphasize action across multiple levels to promote sustainable health changes [25]. In this context, multicomponent interventions are defined as structured approaches that integrate two or more components—such as dietary strategies, physical activity, and behavior change techniques—within the same care setting, guided by established taxonomies such as the Behavior Change Technique Taxonomy (BCTTv1) [26,27], to improve health behaviors and related outcomes [28,29].

Interventions that address these determinants are more attuned to the lived experiences of women with obesity and contribute to more equitable and contextually appropriate care. This study presents the NutrirCom protocol, a multicomponent nutritional intervention designed to provide comprehensive care for women with obesity, alongside the psychological and emotional outcomes observed in one of the intervention arms. This study aimed to describe the methodology and evaluate the effects of a multicomponent nutritional intervention, not focused on caloric restriction, on psychoemotional outcomes.

## 2. Materials and Methods

The study aims to present the NutrirCom protocol, a multicomponent nutritional intervention designed to provide comprehensive care for women with obesity, as well as to describe the psychological and emotional outcomes observed in one of the intervention arms.

### 2.1. Study Design and Setting, Participants, Randomisation and Blinding

This was a prospective, three-arm parallel randomised clinical trial conducted within the primary health care setting of Viçosa (Brazil) between July 2022 and February 2023.

Adult women (aged ≥ 18 and <60 years) with a body mass index (BMI) ≥ 30 kg/m^2^ were eligible for inclusion. The exclusion criteria included current use of antiobesity medication; severe medical or psychiatric conditions; insulin-dependent diabetes; untreated thyroid disorders; pregnancy or lactation; and a history of bariatric surgery. Recruitment was conducted through referrals from the primary health care network, social media outreach, and active searches within primary care units.

Randomisation was performed by two independent researchers via the “random” function in Excel 365, with stratification by BMI. Allocation lists were kept confidential to ensure allocation concealment. This was an open-label study; however, statistical analyses were conducted by a team blinded to group allocation.

No changes to the study methods, eligibility criteria, interventions, or outcomes were made after trial commencement.

### 2.2. Procedures

For Groups 1 and 2, individual sessions were held fortnightly during the first three months and monthly during the subsequent three months [30,31]. For Group 3, individual sessions occurred monthly throughout the six-month intervention period and were combined with one monthly group session. Individual sessions lasted approximately 40 min, whereas group meetings lasted 60 min. All interventions were conducted in person. The protocol was developed by a multidisciplinary and interdisciplinary research team composed of nine nutritionists, two nurses, two psychiatrists, and one psychologist. Nutritional care was delivered by nutritionists, while the multidisciplinary and interdisciplinary team met regularly to discuss cases and plan the interventions.

#### 2.2.1. Group 1 (G1)—Control Group

The control group received an average of nine individual nutritional counselling sessions over six months. A personalised calorie-restricted meal plan was prescribed, developed via Avanutri^®^ software, with the aim of achieving a 5–10% reduction in body weight [30], in addition to nutritional guidance on the basis of the recommendations of the Brazilian Dietary Guidelines (GAPB). Body weight was monitored monthly, with reinforcement of adherence and encouragement to engage in physical activity.

#### 2.2.2. Groups 2 (G2) and 3 (G3)—Intervention Groups

The NutrirCom intervention protocol was developed based on a comprehensive review of scientific studies, national and international guidelines, educational materials, and technical reports related to obesity care. These frameworks were selected and discussed by nutritionists, psychologists, and researchers, guiding the design of the intervention components. The protocol was developed by the authors and does not represent a direct adaptation of a pre-existing model, but rather results from the critical integration of established evidence and frameworks, specifically structured for the context of Primary Health Care.

The intervention was grounded in nutritional counselling sessions informed by person-centred care [32], motivational interviewing [33], the transtheoretical model of behaviour change [34], principles of health communication [35], Non-Violent Communication [36], and complex systems thinking [37]. Nutritional strategies addressed clinical, nutritional, and behavioural constructs commonly observed in women with obesity, including dysfunctional eating behaviours, body dissatisfaction, repeated dieting, low food and interoceptive awareness, low self-compassion, irregular eating patterns, non-adherence to the Brazilian Dietary Guidelines, and unhealthy lifestyles. These elements informed personalised strategies such as SMART goal setting, reflective food diaries, social support enhancement, emotional regulation, cognitive restructuring, self-efficacy reinforcement, and relapse prevention (Supplementary Material S1).

Interventions in Groups G2 and G3 were organised through the selection of specific behaviour change techniques from the BCTTv1 [26,27] (Supplementary Material S2). In this study, the term multicomponent is used according to explicit conceptual and taxonomic criteria, rather than as a synonym for non–weight-centred interventions or the simple combination of techniques. NutrirCom was conceived from its inception as a genuinely multicomponent intervention, grounded in a framework of complexity and person-centred care and structured around four interdependent axes: policy dimensions, core strategies, guiding principles, and health actions.

**Figure 1:**
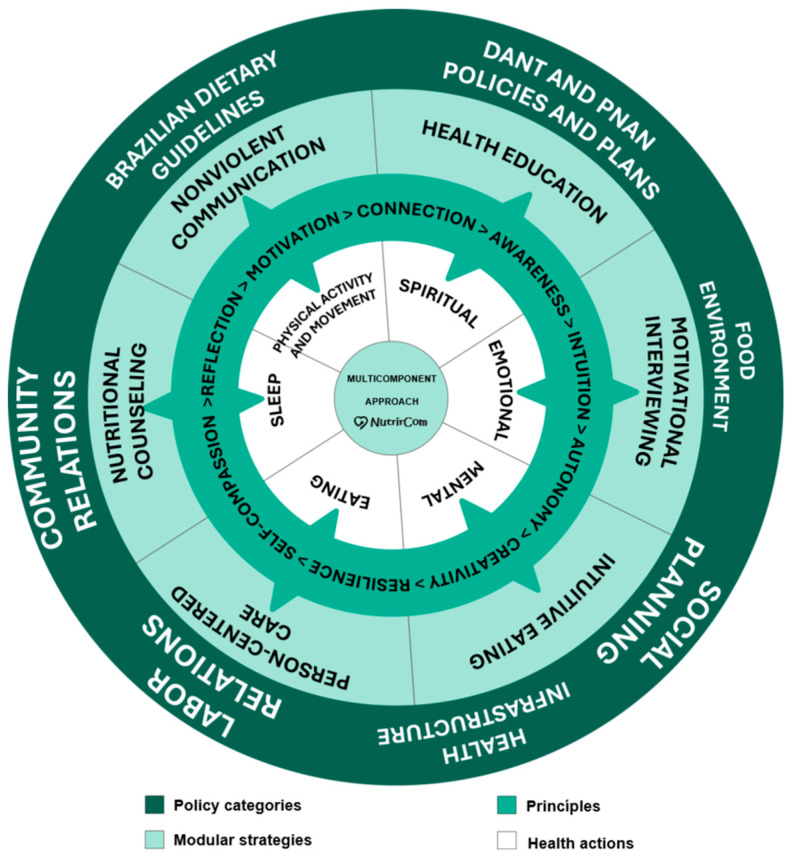
Dimensions of the NutrirCom multicomponent approach. Note: DANT = chronic noncommunicable disease; PNAN = National Food and Nutrition Policy.

The intervention actions were aligned with the Brazilian Dietary Guidelines [38] and incorporated reflective educational activities aimed at strengthening self-care and self-compassion, emphasising internal listening, body acceptance, and a non-judgemental relationship with food and the body. Accordingly, the NutrirCom approach conceptualises eating as a complex, relational practice shaped by symbolic, bodily, and social meanings, enabling an integrated and context-sensitive intervention. The resulting NutrirCom model, illustrated in Figure 1, synthesises the political dimensions, core strategies, guiding principles, and health actions that underpin both the conceptual development and practical implementation of the protocol.

#### 2.2.3. Theoretical Framework of the Intervention

(a) Policy Categories

The treatment of obesity in women requires public policies that go beyond the biomedical perspective, adopting an integrated approach that considers emotional, social, and cultural factors [39]. In addition to promoting social inclusion and combating stigma, it is essential to coordinate actions that integrate mental health, nutritional education, encouragement of physical activity, and social support [40].

The Dietary Guidelines for the Brazilian Population guide conscious and sustainable food choices, valuing local dietary practices and recognising the social determinants of health and the cultural, social, and environmental factors that influence eating behavior [38]. In this context, healthy environments in living, work, and social spaces are essential for the promotion of adequate habits and a healthy lifestyle [40,41,42,43,44,45,46,47,48]. The structure of health services should be multidisciplinary, focusing on nutritional education and prevention, following guidelines such as the Dietary and Nutritional Care Plan (DANT) and the National Food and Nutrition Policy (PNAN), which reinforce the need for favourable food environments and integrated actions to reduce obesity and associated chronic diseases.

Obesity must be understood as a complex condition influenced by structural factors that go beyond individual choices. Socioeconomic, cultural, and environmental contexts shape eating patterns and lifestyles, requiring that public policies be developed in an articulated manner, promoting food and nutrition education, access to healthy foods, suitable spaces for physical activity, and psychosocial support.

At the individual level, these policies can be combined with psychological support strategies and behavioral modifications to promote healthier choices. At the collective level, community engagement and the strengthening of social support networks are essential for creating a health-promoting environment and preventing obesity [49,50].

These policy actions, when aligned with the principles of social planning, have the potential to reduce obesity while promoting health in a more equitable and sustainable way.

(b) Core strategies

The treatment of obesity in women must be comprehensive, encompassing physical, emotional, and psychological well-being. Strategies such as person-centered care, nutritional counselling, nonviolent communication, health education, motivational interviewing, and intuitive eating contribute to more humanised, sustainable care that is aligned with individual needs [33,51,52,53,54].

The NEJM Catalyst emphasises the importance of respecting patients’ preferences, values, and life contexts, highlighting the need for an active partnership between health care professionals and patients in the development of care plans [55]. This person-centered approach is also advocated by Ferraris and De Amicis [56], who define nutritional counselling as a collaborative process in which professionals and patients work together to set goals and develop personalised action plans. The authors highlighted that this strategy is essential for promoting sustainable lifestyle changes and effectively managing chronic diseases, reinforcing the need for care on the basis of active listening, dialogue, and shared responsibility.

(c) Guiding Principles

The intervention is based on guiding principles such as self-compassion, reflection, motivation, connection, awareness, intuition, autonomy, creativity, and resilience. These elements strengthen mental and emotional health, promoting sustainable lifestyle changes. Self-compassion reduces self-criticism; reflection supports the understanding of eating patterns; motivation and connection sustain the change process; awareness and intuition enhance the recognition of bodily signals; autonomy reinforces engagement; creativity allows for individualised adaptations; and resilience contributes to overcoming obstacles. Understanding these principles also supports the monitoring and evaluation of health actions.

(d) Health Actions

Obesity care requires the integration of different health dimensions, such as mental health, nutrition, sleep, physical activity, movement, spirituality, and emotional health. These factors are interrelated and influence both eating behavior and treatment adherence [57].

Balanced eating, combined with practices such as intuitive eating, promotes a healthier relationship with food. Inadequate sleep and physical inactivity increase the risk of obesity, whereas enjoyable and mindful regular physical activity contributes to physical and psychological well-being [16,58].

Spirituality, understood as a source of purpose and connection with what each person considers meaningful, can strengthen emotional coping [59]. According to Puchalski et al. [60], spirituality can be expressed through beliefs, values, or practices and represents an individual’s search for connection and transcendence—whether through relationships with friends, family, nature, work, or animals or through any element the person considers sacred. The development of emotional health and resilience, in turn, helps regulate stress and prevent dysfunctional eating patterns [61].

**Figure 2:**
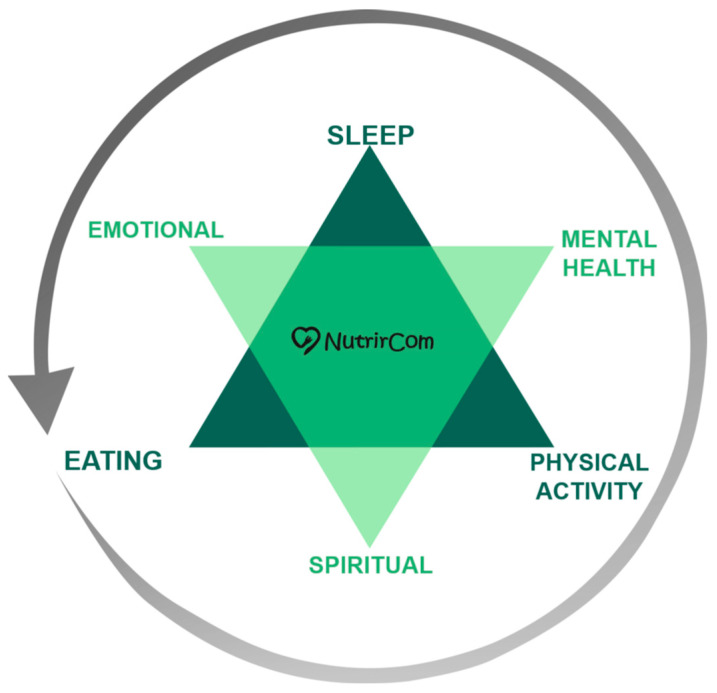
Person-centered care model of the NutrirCom approach.

By integrating these different dimensions, the intervention protocol proposes a person-centered care model that recognises and addresses the multiple influences involved in the development and maintenance of obesity (Figure 2). This proposal aims to offer a sustainable path to health promotion, which is distinct from approaches focused exclusively on caloric restriction. It involves a combination of interventions focused on mental health, nutrition, physical activity/movement, sleep, spirituality, and emotional health, offering a holistic approach to obesity treatment that considers the entirety of the individual.

Table 1 details the planned interventions, which are aligned with the tools and strategies used to achieve the objectives.

For the combined approach (G3), it was acknowledged that social support fosters adherence and enhances outcomes by strengthening interpersonal bonds and encouraging shared responsibility [35,62,63]. The group sessions focused on active listening, Nonviolent Communication (NVC), life narratives, and meaningful goal setting, addressing psychosocial, educational, and behavioural themes aligned with the Nutrition and Food Instruction in Primary Care (Table 2) [64].

### 2.3. Data Collection

Body composition was assessed via anthropometry at the Health Division Laboratory of the Federal University of Viçosa by a trained team following standardised protocols [65]. Body weight was measured via a digital scale (Toledo do Brasil, model 2096PP, 150 kg capacity, São Paulo, Brazil), and height was measured with a wall-mounted stadiometer (Alturexata^®^), enabling BMI calculation (kg/m^2^) and classification according to WHO criteria [66]. Total body fat and android fat distributions were assessed via Dual-energy X-ray absorptiometry (DXA) (Lunar Prodigy Advance, GE Medical Systems Lunar, Milwaukee, WI, USA).

Blood samples were collected via peripheral venepuncture by a certified phlebotomist at a laboratory accredited by the Viçosa Municipal Health Secretariat. Serum cortisol, which is used as a stress indicator, was measured approximately 2 h after the usual waking time following a 12 h fast via an Atellica^®^ CI analyser with Siemens reagents (Espírito Santo, Siemens Healthineers, São Paulo, Brazil).

Subjective stress was assessed via the Adult Stress Symptoms Inventory (ISSL), which was validated by Lipp and Guevara [67] and is based on Selye’s three-phase model [68]. The instrument evaluates 37 physical and 19 psychological symptoms experienced over the previous 24 h, week, and month and demonstrates high internal consistency (α = 0.93; ω = 0.94) and good fit indices (CFI, TLI ≥ 0.9; RMSEA, SRMR ≤ 0.08).

Anxiety and depression were measured via the Hospital Anxiety and Depression Scale (HADS; 14 items), with cut-off scores of 0–7 (unlikely), 8–11 (possible), and 12–21 (probable). The Brazilian version of the HADS has good internal consistency, test–retest reliability (≥0.70), and convergent validity [69,70,71].

Self-compassion was evaluated via the 26-item self-compassion scale (SCS), adapted for Brazil by Souza and Hutz [72], which is scored on a five-point Likert scale, with negatively worded items reverse-coded. The Brazilian version shows high internal consistency (α = 0.92) [73].

As part of the study design, clinical, behavioural, psychosocial, and laboratory variables were collected; however, this article reports outcomes related to anxiety, depression, stress, and self-compassion. Additional assessments included sociodemographic characteristics, blood pressure, sleep quality (PSQI) [74], dietary adherence [75], quality of life (WHOQOL-bref) [76], mindful eating (MEQ) [77], eating behaviours (DEBQ) [78], life satisfaction (Wheel of Life) [79], body image (Stunkard Silhouette Scale) [80], and dietary intake (24 h recall). Laboratory analyses included complete blood count; glucose, insulin, and HbA1c; lipid profile; liver and renal function markers; electrolytes; vitamin D; C-reactive protein (CRP); and cortisol.

**Figure 3:**
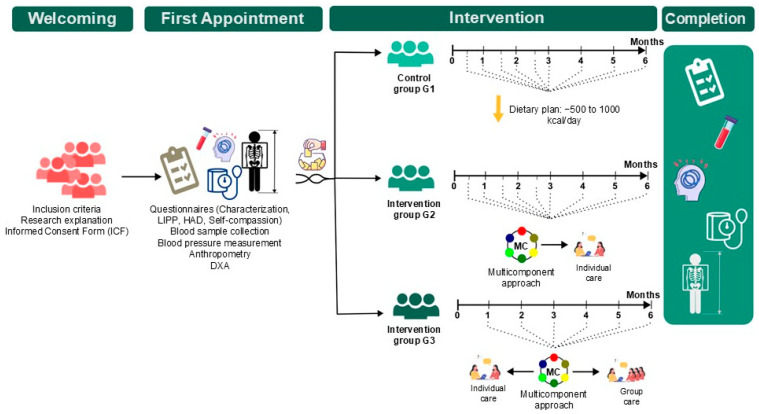
Data collection, randomisation, and intervention process. Note: DXA = Dual-energy X-ray absorptiometry.

A pilot study involving 16 women, independent of the final sample, was conducted to standardise the methodology, test the questionnaires, and train the intervention nutritionists. Figure 3 illustrates the data collection, randomisation, and intervention processes.

### 2.4. Outcomes

The outcomes evaluated in this study included reductions in stress levels, assessed via ISSL and serum cortisol; symptoms of anxiety and depression, assessed via HADS; self-compassion, assessed via the SCS; anthropometric parameters (BMI, waist circumference, waist–hip ratio); body composition (total and android fat, lean mass); and metabolic markers (fasting glucose, complete blood count, lipid profile, liver enzymes, and renal function).

### 2.5. Statistical Analysis

The sample size calculation was performed via Epi Info™ software (version 7.2), and the StatCalc module was used to compare two independent proportions (two population proportions). A 95% confidence level, 80% statistical power, and expected proportions of 41% in the exposed group and 8% in the nonexposed group were adopted on the basis of previous findings in the literature. This procedure yielded a minimum sample size of 78 participants, equivalent to 26 individuals per group when adapted to a three-arm design. The calculation was conducted considering the worst-case scenario between two contrasting conditions, ensuring sufficient power to detect clinically relevant differences between any of the intervention groups and the control group.

To mitigate potential loss to follow-up and maintain adequate power for multiple comparisons, 168 participants were recruited; of these, 89 completed all stages of the study.

The normality of distributions was assessed via the Shapiro–Wilk test and graphical inspection. For variables with nonnormal distributions, bootstrap resampling (1000 resamples) was applied to confirm the robustness of the results. To evaluate within-group changes between the pre- and postintervention periods, paired t tests or Wilcoxon tests were applied to continuous variables according to distributional assumptions.

Between-group comparisons were performed via analysis of covariance (ANCOVA), with the pre-post intervention difference (Δ = post − pre) for each outcome as the dependent variable. Baseline variables that were associated with the intervention (*p* < 0.200) in the exploratory one-way ANOVAs were included as covariates. The homogeneity of regression slopes was verified in advance as a prerequisite for ANCOVA. For variables with nonnormal distributions, model significance was additionally confirmed via bootstrap methods. Post hoc Sidák correction tests were performed for variables that showed between-group differences in the ANCOVA. Statistical analyses were conducted via SPSS version 20.0 (IBM, Armonk, NY, USA), with a 5% significance level (*p* < 0.05).

**Figure 4:**
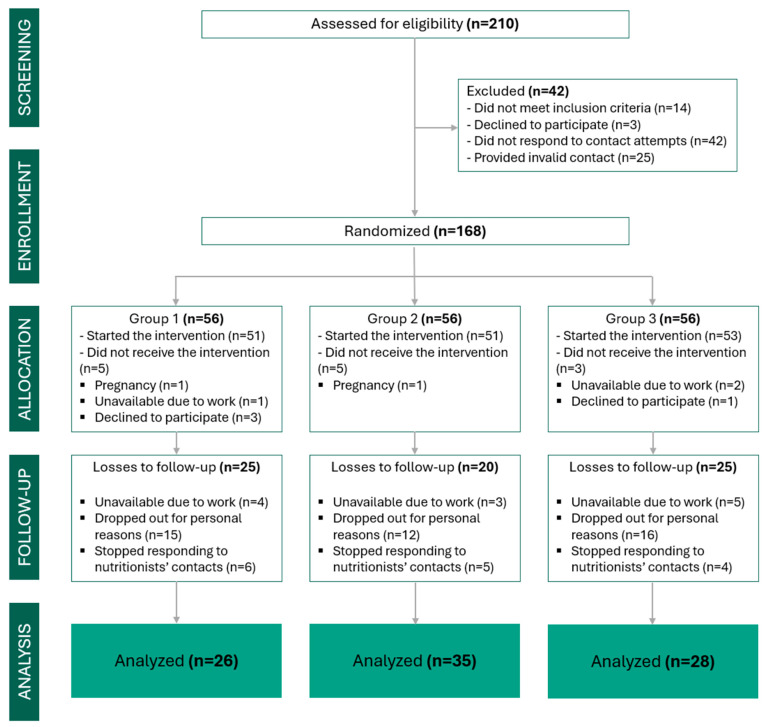
CONSORT flow diagram illustrating the study design and participant progression. It details the number of patients assessed, randomised, allocated to treatment groups, and included in the final analysis.

The recruitment process is illustrated in Figure 4, which presents a flowchart developed in accordance with the CONSORT guidelines for randomised controlled trials with multiple arms.

## 3. Results

Among the 168 women randomised, 89 completed the six-month intervention (G1 = 26; G2 = 35; G3 = 28), corresponding to a retention rate of 53%. Baseline anthropometric, metabolic, and psychosocial characteristics were comparable across groups (Table 3).

The results presented here refer to preliminary data from one arm of the NutrirCom study, which forms part of a broader multicomponent intervention umbrella protocol.

After six months, all groups presented significant reductions in waist circumference and the waist–to–hip ratio, as well as decreases in fasting glucose levels (Table 4). Reductions in total and android body fat assessed by DXA, accompanied by increases in lean mass, were observed across all groups. A significant reduction in BMI was observed only in the hypocaloric control group (G1). No significant between-group differences were detected for anthropometric or metabolic outcomes after adjustment.

With respect to psychosocial outcomes, anxiety decreased significantly in the intervention groups (G2 and G3), with no significant change observed in the control group. Only G2 demonstrated a statistically significant reduction in depressive symptoms. The stress indicators revealed divergent patterns, with increases in psychological stress observed in the control group, whereas consistent reductions in the resistance and exhaustion phases of stress were identified in the intervention groups. Between-group analyses confirmed significant differences for the resistance and exhaustion stress phases (Table 5).

In the ANCOVA adjusted for baseline values and selected covariates, a statistically significant difference was observed between groups for the resistance variable (LIPTOTAL2; *p* = 0.017), indicating differences among at least two groups after adjustment. Post hoc analyses revealed that this difference occurred specifically between Group 1 and Group 2 (*p* = 0.017), with no statistically significant difference between Group 1 and Group 3 (*p* = 0.082) or between Groups 2 and 3. Overall, among the variables that reached significance in the adjusted model, differences occurred predominantly between the control group and the intervention groups, whereas no significant differences were identified between Groups 2 and 3, suggesting similar effects between the two NutrirCom intervention modalities.

For cortisol, ANCOVA revealed an overall difference between the groups (*p* = 0.036). However, multiple comparisons did not reveal statistically significant differences in pairwise analyses (Group 1 vs. Group 2; Group 1 vs. Group 3; Group 2 vs. Group 3), although a trend toward a difference between Groups 2 and 3 was observed (*p* = 0.063). These findings indicate that despite the global significance of the adjusted model, it was not possible to identify robust specific contrasts between groups for this variable. The covariates included in the adjusted model were age, systolic blood pressure, depression, resistance, exhaustion, psychological stress, and physiological stress.

Overall, Groups G2 and G3 demonstrated more consistent improvements in stress, anxiety, depression, and self-compassion indicators than did the control group. These preliminary findings suggest positive effects of the multicomponent approach on psychosocial outcomes in women with obesity. No adverse events were reported.

## 4. Discussion

The intervention groups showed consistent reductions in anxiety and stress levels, as well as increases in self-compassion; however, a statistically significant reduction in depressive symptoms was observed exclusively in Group 2. Although the control group (G1) showed a reduction in BMI, the intervention groups demonstrated broader improvements in body composition, with reductions in body fat and waist circumference, alongside increases in lean mass. These findings highlight the potential of multicomponent interventions to yield both clinical and psychosocial benefits without focusing exclusively on weight loss.

The increase in stress levels observed in the hypocaloric control group is consistent with evidence linking calorie-restricted interventions to heightened psychological stress, increased cognitive burden related to eating, and intensified feelings of guilt and failure, particularly among women.

The assessment of body composition via DXA represents a relevant methodological strength of this study, allowing for a more precise analysis of changes in fat and lean mass compartments beyond variations in BMI. Unlike interventions that predominantly report reductions in body weight, the NutrirCom results demonstrated consistent reductions in total body fat and waist circumference, concomitant with increases in lean mass in the intervention groups. These findings are aligned with studies that emphasise the importance of qualitative body-related outcomes in obesity care [81,82,83]. Previous evidence indicates that multicomponent interventions integrating diet, physical activity, and psychosocial support can promote improvements in body composition independently of large weight reductions, particularly when assessed via higher-accuracy methods such as DXA [83,84,85]. These findings reinforce that an isolated interpretation of BMI may underestimate clinically relevant physiological benefits and support the adoption of approaches that value broader and more sustainable body changes.

The integration of psychological and physiological components constitutes one of the central pillars of the NutrirCom protocol, particularly in the group that combines individual consultations and group sessions. The social support fostered in group sessions (G3) may have contributed to reductions in stress, anxiety, and depressive symptoms through psychobiological mechanisms already described in the literature, such as attenuation of chronic activation of the hypothalamic–pituitary–adrenal axis and reductions in cortisol levels, factors directly associated with central fat accumulation and metabolic alterations [60,61,85,86]. Previous studies have demonstrated that interventions based on social support and a sense of belonging are associated with improved emotional regulation, greater adherence to healthy behaviors, and more favourable physiological responses, including improvements in body composition and glucose metabolism [35,64,81,87]. In this context, the results observed in the present study suggest that strengthening social bonds and creating emotionally safe environments not only enhance psychological well-being but also may act as mediators of relevant physiological changes in obesity treatment.

The dropout rate observed in this study (approximately 47%) should be interpreted in light of the primary health care context and the complexity of obesity care, particularly in longitudinal interventions. Structural barriers, such as transportation difficulties, family and work demands, and psychosocial vulnerabilities frequently associated with obesity in women, may have influenced the continuity of follow-up. Nevertheless, the study was completed with 89 participants, a number exceeding the previously estimated minimum sample size (*n* = 78), ensuring adequate statistical power to detect clinically relevant differences between groups and supporting the robustness of the analyses performed.

Several approaches used in obesity care—such as mindful eating, acceptance-based interventions, Health at Every Size–oriented frameworks, motivational interviewing, and cognitive–behavioural models—are generally grounded in specific theoretical or methodological strategies. These approaches are often not centred on caloric restriction and are aligned with a more humanised perspective of care. However, when examined using explicit conceptual and methodological criteria, these proposals do not, in isolation, constitute structured multicomponent interventions.

In the present study, the NutrirCom protocol differs by operationalising these strategies within an explicitly multicomponent model, developed for the context of Primary Health Care and grounded in well-defined conceptual and taxonomic criteria. Unlike interventions in which multiple techniques are primarily combined to optimise weight loss, NutrirCom prioritises outcomes such as psychoemotional health, self-compassion, eating autonomy, and contextual determinants of behaviour. The protocol is grounded in the Brazilian Dietary Guidelines, the BCTTv1, principles of Nonviolent Communication, and complex systems thinking, enabling a structured, integrated, and context-sensitive intervention. In addition, the integration of individual counselling with structured group sessions delivered by a multidisciplinary team within the public health system enhances the feasibility, scalability, and real-world applicability of NutrirCom.

The protocol was structured on the basis of frameworks recognising obesity as a multifactorial condition, such as Edgar Morin’s theory of complex thought, which emphasises the need for integrated approaches capable of embracing the complexity of human and social phenomena [88]. The development of the intervention integrated multiple dimensions of care, overcoming fragmented and reductionist views. It involves an interdisciplinary team that combines scientific evidence with clinical practice knowledge, which is grounded in active listening, person-centred care, and the promotion of holistic health [36,72,73].

Traditional approaches based on hypocaloric diet prescriptions have shown limitations when faced with the complexity of obesity and may increase the risk of eating disorders, binge eating, body dissatisfaction, and emotional distress [89,90]. In contrast, NutrirCom fosters reconnection with internal hunger and satiety cues, avoids the moralisation of food, and strengthens eating autonomy, in alignment with the Brazilian Dietary Guidelines [91]. The increase in self-compassion levels observed in the intervention groups supports the effectiveness of strategies that promote a more respectful relationship with the body and food [92,93,94].

Importantly, evidence-based obesity treatment encompasses a broad range of approaches beyond traditional hypocaloric diet prescriptions, including structured behavioral programs, acceptance- and mindfulness-based interventions, lifestyle change strategies grounded in cognitive-behavioral models, telemedicine-mediated interventions, and integrated multidisciplinary care [26,33,34,60,61,88,91]. Several studies already cited in this discussion demonstrate that these approaches can produce clinically and psychoemotionally relevant benefits when adapted to context, individual needs, and different care formats, including in-person and remote modalities [81,82,83,84,95,96,97,98]. The results of the present study do not invalidate such evidence-based models but rather underscore the importance of how different therapeutic components are articulated and prioritised. In this sense, the NutrirCom protocol does not position itself in opposition to existing interventions; instead, it engages with this body of knowledge by systematically integrating nutritional, behavioral, emotional, and contextual dimensions within a non-weight-centered approach, aiming to reduce potential negative psychoemotional impacts without compromising clinical effectiveness.

The reduction in anxiety observed in groups G2 and G3 may be associated with the creation of an emotionally supportive environment, the emphasis on body acceptance, and the recognition of emotional eating patterns. Active listening and the use of elements inspired by NVC strengthened adherence and engagement, increased emotional awareness, and reduced feelings of guilt [36,99,100]. Anxiety has often been linked to obesity [101,102]. Strategies aimed at reducing weight-related stigma, promoting body image acceptance, and encouraging guilt-free eating may have contributed to the reduction in these symptoms, fostering a more emotionally welcoming environment.

The combination of individual consultations and group sessions in Group 3 proved feasible and was associated with psychosocial improvements similar to those observed in Group 2, with no evidence of statistically significant superiority between the interventions.

In this context, the multicomponent approach helps identify episodes of emotional eating and loss of control, which may reduce the anxiety associated with following a dietary plan and the feeling of needing to “report back” to the professional. This awareness supports a more reflective and autonomous relationship with food, promoting greater emotional balance and more effective adherence to nutritional guidance [103], supported by techniques of emotion identification and naming, as proposed by NVC.

The findings of this study regarding the reduction in stress and depressive symptoms among participants receiving the multicomponent intervention are consistent with evidence previously reported in the literature. Studies such as those of Duncan et al. [81] and Sabatini et al. [95] also reported significant improvements in psychoemotional outcomes following interventions that integrated various care strategies, including nutritional counselling, psychosocial support, and the promotion of physical activity.

Moreover, the study by Dalen et al. [82], which applied mindfulness techniques to adults with obesity, demonstrated beneficial effects on reducing stress and depressive symptoms, reinforcing the role of integrated approaches in emotional care. Similarly, Ulian et al. [84] and Gonçalves et al. [100] reported reductions in psychological distress and depressive symptoms in obese women who underwent interventions addressing emotional, behavioural, and social aspects, findings that corroborate the results of the present study. This body of evidence strengthens the understanding that multicomponent, person-centred care can promote not only physical benefits but also meaningful improvements in the mental health of obese women.

The actions proposed by the protocol, such as mindful eating, active listening, spirituality, sleep promotion, and encouragement of enjoyable physical activity, targeted multiple dimensions of care and were fundamental to improving participants’ quality of life. The integration of these aspects is associated with greater adherence to behaviour change and the sustainability of the outcomes achieved [61]. The collective sessions (G3) also stood out, promoting a sense of belonging, bonding, and shared responsibility among participants, elements already recognised as essential for the success of public health interventions [35,64].

Other authors, when applying behavioural interventions that place minimal demands on professionals and participants, such as unsupervised low- to moderate-intensity exercises (e.g., brisk walking), sustainable dietary changes, and cognitive–behavioural techniques, also reported positive effects on depressive symptoms, both in vulnerable populations and in groups with greater access to health care resources [83,96,97,98]. These findings reinforce that integrated and accessible interventions, such as NutrirCom, can play a significant role in promoting mental and physical health, especially in challenging contexts such as primary health care.

The NutrirCom protocol was built upon solid theoretical and methodological foundations, such as the BCTTv1 [26], the Dietary Guidelines for the Brazilian Population [38], motivational interviewing [33], NVC [35], and intuitive eating [104]. This framework enabled the development of a replicable, accessible, and low-cost methodology adaptable to diverse primary care contexts. Personalised care, active listening, and shared responsibility between professionals and users enhanced the effectiveness of the intervention, offering viable pathways for public health policies aimed at improving care for women with obesity.

Among the dimensions addressed in the intervention protocol, we highlight mental and emotional health, the importance of physical activity and movement, and sleep and spirituality. Sleep deprivation, associated with hormonal changes such as increased ghrelin and cortisol and decreased leptin, contributes to weight gain and metabolic alterations [85,86]. Therefore, promoting adequate sleep is recommended as an essential component of obesity care. The integration of multiple domains, including nutrition, sleep, movement, spirituality, and mental health, enhances the effects of interventions and supports sustainable changes [61]. Spirituality, in turn, can support a sense of purpose and motivation for adopting healthy behaviours [60,87].

Despite recent advances, studies that structurally evaluate multicomponent interventions that do not focus on weight loss as the primary goal are lacking. The literature suggests that combining nutritional strategies, emotional support, physical activity promotion, and strengthening of social bonds can lead to sustainable benefits even in vulnerable populations [81,105,106]. NutrirCom helps to fill this gap by offering a robust intervention applicable to the health system, showing positive impacts on both physical and emotional indicators. Future studies should further analyse the contribution of each component of the intervention and its potential for large-scale implementation.

Among the strengths of this study are the randomised clinical trial design, the use of validated instruments, and the assessment of body composition through DXA. The intervention demonstrated operational feasibility, adherence to comprehensive care guidelines, and potential for scalability.

Several limitations should be considered when interpreting the results. The possibility of selection bias cannot be excluded, as participants who remained in the study may have demonstrated greater motivation, availability, or engagement with health care. However, randomisation with allocation concealment, baseline comparability between groups, and the use of validated instruments, together with covariate-adjusted analyses and bootstrap resampling procedures, help to mitigate these potential biases and strengthen the internal validity of the study. In addition, the multicomponent nature of the intervention limits the attribution of effects to isolated components, a characteristic inherent to complex, person-centred interventions.

## 5. Conclusions

The NutrirCom intervention was effective in promoting emotional, clinical, and behavioural improvements in women with obesity, including reductions in stress, anxiety, and depression, alongside fostering self-compassion. The strategies employed also had a positive effect on body composition and biochemical markers. Its primary innovation lies in the integration of nutritional, psychological, and community dimensions, with a focus on mental health. Given the significance of obesity and mental disorders in women as critical public health issues, the NutrirCom contributes to more comprehensive care that is sensitive to the complexity of these contexts. The results underscore the need for public policies that promote the transdisciplinary training of professionals, enabling them to implement person-centred approaches and foster sustainable improvements in both physical and emotional health.

These findings should be interpreted with caution and underscore the need for future studies with greater statistical power to further elucidate the specific effects of each intervention component.

## Figures and Tables

**Table 1:** Themes, Strategies, and Tools of the NutrirCom Approach.

Themes	Strategies	Tools
Food quality and choices	Choosing foods	Brazilian Dietary Guidelines (GAPB) healthy eating steps
	Food combination and portioning	GAPB plate elements (portion size, quantity, quality, and variety)
	Food preparation	Recipes
	Meal planning	Shopping planning, menus, and food prep techniques
	Stimulating cooking skills	Recipes, home gardening, use of natural seasonings, functional foods/fibre
The act of eating	Eating regularly and in appropriate environments	GAPB healthy eating steps
	Mindful eating—being present during meals	Tools to promote mindful eating
	Recognising hunger, satiety, and satisfaction cues	Intuitive Eating principles
	Recognising emotional eating	Strategies to manage emotional eating NVC list of feelings and needs
	Pleasure in eating and sharing meals	Avoiding food dichotomy Reflective food diary
Self-connection	Relaxation and reducing anxiety	Breathing exercises NVC list of feelings and needs
	Presence	Conscious screen time use
	Identifying emotions and feelings	NVC list of feelings and needs
	Self-monitoring	Reflective food diary
	Understanding the connection between emotions and eating behavior	Reflective food diary Strategies to manage emotional eating Spirituality activities
Self-care and self-acceptance	Positive body image	Respect for the body Self-compassion scale Conscious use of social media
Healthy lifestyle (individual and collective)—the territory	Movement/physical activity	Encouraging enjoyable physical activity
	Quality sleep	Sleep hygiene
	Leisure and family/social connection	Life Wheel Promoting leisure and family relationships Improved relationship with work Requesting needs

GAPB = Brazilian Dietary Guidelines; NVC = Nonviolent Communication.

**Table 2:** Themes and Objectives of the Group Sessions—G3 NutrirCom.

Session	Theme	General Aims	Specific Aims
1	Welcome to the NutrirCom Journey	Cocreate with the women the motivations for engaging in the NutrirCom journey	Welcome the participants and learn about their intentions, motivations, and aspirations for the journey. Establish group agreements. Deliver the materials and explain how they will be supported.
2	Me and My Inner Child	Strengthen the inner child to support changes in eating behavior	Explore beliefs and habits formed in childhood. Work on the child’s positive aspects to support behavior change.
3	Reflection on the Act and Manner of Eating	Reflect on food choices and how one eats	Encourage awareness of flavours, textures, and elements of food. Reflect on what the body expresses when tasting food.
4	My Choice Cares for the WHOLE	Strengthen body self-awareness	Encourage self-care with the body. Understand the wisdom of the body. The body as an expression of the spirit. The relationship between body and food: hunger and satiety.
5	Knowledge and Flavours of the Kitchen: The Art of Cooking	Reflect on the act of cooking and food preparation	Conduct tasting of a vegetable-rich dish. Practice mindful eating. Explore the challenges and possibilities of cooking.
6	Real and Individual Paths	Reflect on the challenges and potentials for transformation in the dimensions of care	Reflect individually and collectively. Develop strategies for challenges experienced collectively.
7	My Journey, My Life	Evaluate the impact of the journey on women’s health	Identify the most effective activities. Understand the challenges and potential of group work. Evaluate the journey.

**Table 3:** Baseline anthropometric and metabolic indicators of the women who participated in the intervention.

	Group 1	Group 2	Group 3	*p* Value
Women (*n*)	26	35	28	
Age (years)	40.8 (±11.7)	39.2 (±9.3)	35.7 (±11.0)	0.198
**Biochemical and adiposity parameters**				
BMI (kg/m^2^)	36.7 (±5)	37.6 (±7.9)	36.6 (±4.2)	0.741
Neck circumference (cm)	36.0 (35; 38)	36.5 (35; 38)	36.0 (35; 38)	0.894
Waist circumference (cm)	111.0 (±11.4)	111.6 (±16.5)	108.1 (±11.6)	0.573
Waist-to-hip ratio	0.9 (0.9; 1.0)	0.9 (0.9; 1.0)	0.9 (0.8; 1.0)	0.496
SBP	119.4 (±12)	115.0 (±13.2)	121.3 (±12.5)	0.150
DBP	80.3 (±8.9)	78.7 (±13)	77.5 (±8.8)	0.658
Fasting glucose (mg/dL)	84.0 (81; 91)	85.0 (82; 91)	85.0 (81; 88)	0.940
HOMA-IR	3.7 (2.2; 5.1)	3.1 (2.3; 5.2)	3.7 (2.3; 5.5)	0.900
HbA1c (%)	5.5 (5.1; 5.6)	5.3 (4.9; 5.6)	5.3 (5.2; 5.6)	0.595
Total cholesterol (mg/dL)	180.6 (±32)	179.4 (±33.4)	184.2 (±37.8)	0.859
LDL (mg/dL)	106.9 (±30)	108.8 (±31.3)	109.4 (±32.9)	0.956
HDL (mg/dL)	50.4 (±8.5)	49.5 (±6.5)	49.9 (±6.5)	0.876
Triglycerides (mg/dL)	87.0 (74; 127)	98.0 (81; 130)	103.0 (72; 157)	0.618
Body fat (%)	48.9 (±4.1)	49.5 (±4.9)	49.8 (±4.2)	0.773
Android fat (%)	4.0 (3.0; 4.0)	4.0 (3.0; 4.0)	4.0 (3.0; 5.0)	0.669
Lean mass (%)	48.0 (±4)	47.5 (±4.7)	47.4 (±4.3)	0.853
**Stress/Depression**				
Anxiety	9.5 (±4.7)	11.3 (±4.3)	4.3 (±10.1)	0.294
Depression	7.5 (±3.3)	9.1 (±4.1)	7.4 (±3.7)	0.146
Alertness	4.0 (1.0; 6.0)	4.0 (3.0; 6.0)	3.5 (2.0; 5.5)	0.511
Resistance	4.1 (±3.6)	7.4 (±3.0)	6.5 (±3.8)	0.002
Exhaustion	4.9 (±4.7)	7.9 (±5.2)	7.5 (±5.7)	0.077
Physiological stress	7.6 (±6.1)	10.9 (±6.3)	10.3 (±6.5)	0.115
Psychological stress	5.2 (±4.9)	8.8 (±4.8)	8.5 (±5.6)	0.020
Baseline cortisol	15.4 (12.0; 20.0)	13.0 (10.1; 19.9)	12.7 (9.6; 18.6)	0.503

Data are *n*; mean (±; SD); 95% confidence interval. BMI = Body mass index; kg = kilogram; m^2^ = square meter; cm = centimeter; mg/dL = milligrams per deciliter; SBP = systolic blood pressure/DBP = diastolic blood pressure; HOMA-IR = homeostatic model assessment of insulin resistance; HbA1c = glycated hemoglobin; LDL = low-density lipoprotein; HDL = high-density lipoprotein.

**Table 4:** Anthropometric, biochemical, and adiposity parameters at baseline and after 6 months in the intervention group.

	Group 1 (*n* = 26)			Group 2 (*n* = 35)			Group 3 (*n* = 28)			*p* Value **
	Baseline	Final	*p* Value *	Baseline	Final	*p* Value *	Baseline	Final	*p* Value *	
BMI (kg/m^2^)	36.7 (5.0)	35.4 (5.4)	0.003	33.6 (31.7; 42.8)	34.0 (30.8; 43.6)	0.128	36.5 (4.2)	35.8 (4.1)	0.150	0.706
NC (cm)	36.8 (3.2)	36.4 (3.1)	0.240	37.2 (3.3)	36.0 (3.3)	<0.001	36.5 (3.38)	35.7 (2.5)	0.109	0.113
WC (cm)	111.0 (11.4)	100.5 (10.6)	<0.001	111.6 (16.5)	101.7 (16.7)	<0.001	108.1 (11.6)	100.5 (12.4)	<0.001	0.562
Waist-to-hip ratio	0.9 (0.1)	0.9 (0.1)	0.003	0.9 (0.9; 1)	0.9 (0.8; 0.9)	<0.001	0.9 (0.1)	0.9 (0.1)	<0.001	0.786
SBP	119.4 (12.0)	120.7 (12.4)	0.469	115.1 (13.2)	112.6 (15.3)	0.311	121.3 (12.5)	119.4 (15.0)	0.424	0.470
DBP	80.3 (8.9)	77.8 (9.7)	0.231	78.7 (13.0)	75.5 (9.5)	0.094	77.5 (8.8)	78.6 (11.9)	0.508	0.087
Fasting glucose (mg/dL)	84.0 (81.0; 91.0)	78.0 (76.0; 79.0)	<0.001	85.0 (82.0; 91.0)	80.0 (75.0; 85.0)	<0.001	85.0 (81.0; 88.0)	81.0 (76.0; 88.0)	0.009	0.499
HOMA-IR	4.1 (2.9)	3.2 (1.3)	0.076	3.1 (2.3; 5.2)	2.8 (2.0; 5.2)	<0.001	4.3 (2.7)	4.1 (0.3)	0.677	0.696
HbA1c (%)	5.5 (5.1; 5.6)	5.3 (5.1; 5.6)	0.233	5.3 (4.9; 5.6)	5.4 (5.1; 5.6)	0.073	5.3 (5.2; 5.6)	5.4 (5.0; 5.7)	0.897	0.666
Total cholesterol (mg/dL)	180.6 (32.0)	186.8 (33.5)	0.295	177.0 (150; 193)	170.0 (156; 198)	0.533	184.8 (38.4)	182.9 (40.2)	0.748	0.661
LDL (mg/dL)	106.9 (30.0)	115.4 (25.3)	0.135	108.8 (82.0)	110.4 (26.9)	0.613	110.2 (33.2)	108.0 (36.3)	0.747	0.109
HDL (mg/dL)	50.4 (8.5)	50.9 (9.9)	0.704	49.5 (6.5)	49.4 (6.0)	0.920	49.8 (6.6)	49.7 (7.5)	0.882	0.870
Triglycerides (mg/dL)	116.1 (73.3)	104.3 (46.0)	0.318	105.9 (36.1)	89.0 (31.4)	0.018	124.6 (63.8)	113.6 (57.5)	0.437	0.923
Body fat (%)	48.9 (4.1)	47.4 (4.4)	0.002	49.5 (5.0)	48.3 (5.2)	0.026	49.8 (4.2)	48.3 (4.4)	0.001	0.752
Android fat (%)	4.0 (3.0; 4.0)	3.0 (3.0; 4.0)	0.002	4.0 (3.0; 4.0)	3.0 (3.0; 4.0)	0.003	4.0 (3.0; 5.0)	3.0 (3.0; 5.0)	0.001	0.205
Lean mass (%)	48.0 (4.0)	49.1 (4.3)	0.002	47.4 (4.8)	48.5 (5.0)	0.042	47.4 (4.3)	48.7 (4.5)	0.006	0.711

Data are *n*; mean (±; SD); 95% confidence interval. BMI = body mass index; kg = kilogram; m^2^ = square meter; cm = centimeter; mg/dL = milligrams per deciliter; NC = neck circumference; WC = waist circumference; SBP = systolic blood pressure/DBP = diastolic blood pressure; HOMA-IR = homeostatic model assessment of insulin resistance; HbA1c = glycated hemoglobin; LDL = low-density lipoprotein; HDL = high-density lipoprotein. * Paired *t* test; ** ANCOVA.

**Table 5:** Stress, anxiety, and depression markers at baseline and after 6 months in the intervention group.

	Group 1 (*n* = 26)			Group 2 (*n* = 35)			Group 3 (*n* = 28)			*p* Value **
	Baseline	Final	*p* Value *	Baseline	Final	*p* Value *	Baseline	Final	*p* Value *	
**Stress/Depression**										
Anxiety	9.5 (±4.7)	8.5 (±4.8)	0.134	11.3 (±4.3)	8.3 (±4.1)	0.002	10.3 (±4.3)	8.6 (±4.9)	0.005	0.876
Depression	7.5 (±3.3)	6.7 (±3.7)	0.206	9.1 (±4.2)	7.0 (±3.8)	0.023	7.4 (±3.8)	6.6 (±4.0)	0.125	0.673
Alertness	4.0 (1.0; 6.0)	3.0 (2.0; 6.0)	0.651	4.0 (3.0; 6.0)	3.0 (1.0; 5.0)	0.178	3.5 (2.0; 5.5)	2.0 (0.5; 5.0)	0.014	0.224
Resistance	4.1 (±3.6)	7.3(±3.6)	<0.001	7.4 (±3.0)	4.9 (±3.6)	0.005	6.5 (±3.8)	5.0 (±3.8)	0.019	0.017
Exhaustion	4.9 (±4.7)	8.0 (±4.2)	<0.001	7. 9 (±5.2)	5.2 (±4.2)	0.025	7.5 (±5.7)	5.0 (±4.4)	0.009	0.003
Physiological stress	7.9 (±6.0)	11.6 (±6.5)	0.011	10.9 (±6.3)	8.2 (±6.0)	0.075	10.3 (±6.5)	7.9 (±6.2)	0.002	0.013
Psychological stress	5.4 (±4.9)	8.0 (±4.5)	0.003	8.8 (±4.8)	5.9 (±4.9)	0.024	8.5 (±5.6)	5.7 (±5.1)	0.001	0.052
Baseline cortisol (µg/dL)	15.4 (11.5; 20.4)	11.9 (9.0; 14.6)	0.015	13.1 (10.1; 19.9)	10.3 (9.1; 16.3)	0.002	13.0 (9.5; 18.3)	11.2 (8.0; 16.3)	0.194	0.036
Self-compassion	3.1 (±0.7)	2.9 (±0.6)	0.159	2.6 (±0.7)	3.4 (±0.7)	<0.001	2.7 (±0.6)	3.6 (±0.84)	<0.001	<0.001

Data are *n*; mean (±; SD); 95% confidence interval. µg/dL = micrograms per deciliter. * Paired *t* test; ** ANCOVA.

## Data Availability

The data presented in this study are available from the corresponding author upon reasonable request. Restrictions apply because the dataset contains sensitive personal and health information from clinical trial participants, and access must comply with ethical and privacy regulations.

## Supplementary Materials

The following supporting information can be downloaded at: https://www.mdpi.com/article/10.3390/nu18030414/s1, Supplementary Material S1: Flowchart of Individual Sessions—Group 2 (NutrirCom) and Group 3 (NutrirCom + Social Support). Supplementary Material S2: Correspondence between NutrirCom intervention components and Behaviour Change Technique Taxonomy version 1 (BCTTv1); Supplementary Material S3: Checklist for Reporting of Multi-Arm Parallel-Group Randomized Trials: Extension of the CONSORT 2010 Statement.

## Abbreviations

The following abbreviations are used in this manuscript:

BCTTv1	Behavior Change Technique Taxonomy Version 1
BMI	Body Mass Index
CFI	Comparative Fit Index
DANT	Chronic Noncommunicable Disease
DBP	Diastolic Blood Pressure
DEBQ	Dutch Eating Behavior Questionnaire
DXA	Dual-energy X-ray Absorptiometry
G1	Group 1 (Control)
G2	Group 2 (Individual intervention)
G3	Group 3 (Individual + group intervention)
GAPB	Brazilian Dietary Guidelines
HADS	Hospital Anxiety and Depression Scale
HbA1c	Glycated Hemoglobin
HDL	High-Density Lipoprotein
HOMA-IR	Homeostatic Model Assessment of Insulin Resistance
ISSL	Adult Stress Symptoms Inventory
LDL	Low-Density Lipoprotein
MEQ	Mindful Eating Questionnaire
NVC	Nonviolent Communication
PNAN	National Food and Nutrition Policy
PSQI	Pittsburgh Sleep Quality Index
R24h	24 hour Dietary Recall
SBP	Systolic Blood Pressure
SCS	Self-Compassion Scale
SPSS	Statistical Package for the Social Sciences
SRMR	Standardised Root Mean Square Residual
TLI	Tucker–Lewis Index
WHOQOL	World Health Organisation Quality of Life Assessment
