# Supplementary material for: Multicomponent Nutritional Approach (NutrirCom) and Its Effects on Anthropometric, Metabolic, and Psychoemotional Outcomes in Women with Obesity: A Three-Arm Randomized Clinical Trial
Source: Nutrients. 2026 Jan 27;18(3):414. doi: 10.3390/nu18030414 (PMC12899254; doi:10.3390/nu18030414)
Supplement: Supplementary file 1 [file nutrients-18-00414-s001.zip › nutrients-4024156-supplementary.pdf]

Supplementary Material S1

Flowchart of Individual Sessions – Group 2 (NutrirCom)

Description:
This flowchart illustrates the sequence, thematic focus, and assessment tools used across the ten individual NutrirCom sessions delivered to Group 2 over six months.

Session structure and content:

Session 1

- Anthropometry
- Anamnesis
- 24-hour dietary recall (R24h)
- Assessment of dietary practices (Brazilian Dietary Guidelines – GAPB)
- Wheel of Life
- Sleep quality index
- Anxiety and depression scales
- Introduction to the "10 Steps of the Brazilian Dietary Guidelines"

Session 2

- Follow-up anamnesis
- Construction of the Strategic Nutritional Plan (SNP)
- Co-creation of participant strategies
- Reflective food diary (optional)
- Quality of life scale

Session 3

- Review of the Strategic Nutritional Plan
- Reflective food diary

Session 4

- Hunger, satiety, and satisfaction
- Intuitive eating applied to the Strategic Nutritional Plan

Session 5

- Plate composition (portioning, quantity, quality, and variety)

- Strategic Nutritional Plan

Session 6

- Recipes
- Water intake guidance
- Shopping planning and meal organization
- Anthropometry
- Strategic Nutritional Plan

Session 7

- Review of progress in the Strategic Nutritional Plan
- Discussion on ultra-processed foods, sugars, sweets, and animal fats

Session 8

- Body respect
- Strategic Nutritional Plan (self-care focus)
- Movement and physical activity
- Sleep quality

Session 9

- Identification of perceived vulnerabilities
- Positive reinforcement
- Relapse prevention plan
- Wheel of Life
- Anthropometry

Session 10

- 24-hour dietary recall (R24h)
- Reassessment of dietary practices (GAPB)
- Anxiety and depression scales
- Quality of life scale
- Sleep quality index
- Self-reflective message

Support Materials Used During Individual Sessions – Group 2

Materials and thematic supports:

- Session 1: List of feelings and needs; Bristol stool scale; explanation of biochemical tests and identification of nutritional deficiencies
- Session 4: Mindful eating practices
- Session 5: Plate composition guidance
- Session 6: Recipes; water intake guidance; shopping planning; freezing techniques
- Session 7: Recipes; use of natural seasonings and functional foods
- Session 8: Body respect; sleep hygiene

Additional cross-cutting materials:

- Emotional eating management
- Nutritional guidance for dyslipidemia, constipation, and diabetes
- Emotional regulation strategies addressing anger, guilt, and shame

Transversal components applied in all sessions:

- Welcoming and active listening
- Check-in (breathing exercise, identification of daily feelings and needs)
- Check-out (reflection on emotional state at session closure)
- Continuous recording in the field diary by the interventionist

Flowchart of Individual Sessions – Group 3 (NutrirCom + Social Support)

Description:
This flowchart presents the individual sessions delivered monthly to Group 3, combined with structured group meetings focused on social support.

Session structure and content:

Session 1

- Anthropometry
- Anamnesis
- 24-hour dietary recall (R24h)
- Assessment of dietary practices (GAPB)
- Wheel of Life
- Sleep quality index
- Introduction to the "10 Steps of the Brazilian Dietary Guidelines"

Session 2

- Construction of the Strategic Nutritional Plan (SNP)
- Co-creation of participant strategies
- Reflective food diary (optional)
- Quality of life scale

Session 3

- Review of the Strategic Nutritional Plan
- Reflective food diary
- Hunger, satiety, and satisfaction
- Intuitive eating applied to the Strategic Nutritional Plan

Session 4

- Plate composition (portioning, quantity, quality, and variety)
- Recipes
- Water intake
- Shopping planning and meal organization
- Anthropometry

- Strategic Nutritional Plan

Session 5

- Review of progress in the Strategic Nutritional Plan
- Ultra-processed foods, sugars, sweets, and animal fats
- Recipes
- Body respect

Session 6

- 24-hour dietary recall (R24h)
- Movement and physical activity
- Sleep quality
- Assessment of dietary practices (GAPB)
- Anxiety and depression scales
- Sleep hygiene and self-care within the Strategic Nutritional Plan

Session 7

- Identification of perceived vulnerabilities
- Positive reinforcement
- Quality of life scale
- Self-reflective message
- Anthropometry
- Relapse prevention plan
- Wheel of Life

Support Materials Used During Individual Sessions – Group 3

Materials and thematic supports:

- Session 1: List of feelings and needs; Bristol stool scale; explanation of biochemical tests and identification of nutritional deficiencies
- Session 3: Mindful eating practices
- Session 4: Plate composition; recipes; water intake guidance; shopping planning; freezing techniques
- Session 5: Recipes; natural seasonings and functional foods; body respect; sleep hygiene
- Session 6: Sleep hygiene

Additional cross-cutting materials:

- Emotional eating management
- Nutritional guidance for dyslipidemia, constipation, and diabetes
- Emotional regulation strategies addressing anger, guilt, and shame

Transversal components applied in all sessions:

- Welcoming and active listening
- Check-in (breathing exercise, recognition of feelings and needs)
- Check-out (reflection on emotional state at session closure)
- Continuous recording in the researcher’s field diary

Themes and Objectives of Group Sessions – Group 3 (Social Support)

Session 1 – Welcome to the NutrirCom Journey
General objective: Co-create motivations for engaging in the NutrirCom journey
Specific objectives:

- Welcome participants
- Identify intentions and expectations
- Establish group agreements
- Provide and explain support materials

Session 2 – Me and My Inner Child
General objective: Strengthen the inner child to support eating behavior change
Specific objectives:

- Explore childhood beliefs and habits
- Reinforce positive internal resources

Session 3 – Reflection on the Act and Manner of Eating
General objective: Reflect on food choices and eating practices
Specific objectives:

- Increase sensory awareness of food
- Reflect on bodily responses during eating

Session 4 – My Choice Cares for the Whole
General objective: Strengthen body self-awareness
Specific objectives:

- Foster body self-care
- Explore hunger and satiety
- Understand the body as an integrated whole

Session 5 – Knowledge and Flavors of the Kitchen: The Art of Cooking
General objective: Reflect on cooking and food preparation
Specific objectives:

- Practice mindful eating
- Taste a vegetable-rich dish
- Explore challenges and possibilities of cooking

Session 6 – Real and Individual Paths
General objective: Reflect on challenges and potentials for transformation
Specific objectives:

- Individual and collective reflection
- Co-construction of coping strategies

Session 7 – My Journey, My Life
General objective: Evaluate the impact of the NutrirCom journey
Specific objectives:

- Identify effective activities
- Assess challenges and strengths of group work
- Evaluate the overall journey

Supplementary Material S2

Correspondence between NutrirCom intervention components and Behaviour Change Technique Taxonomy version 1 (BCTTv1)

BCT technique (BCTTv1)	NutrirCom Multicomponent Intervention
Behavioural goal setting	Establishment of SMART goals
Action planning	Development of an individualised nutrition plan and strategies to overcome barriers
Review of behavioural goals	Continuous re-evaluation of goals across sessions
Feedback on behaviour	Discussion of progress, challenges, and strategies during sessions
Self-monitoring of behaviour	Use of a reflective food diary
Restructuring the physical/social environment	Adaptation of dietary routines and the home environment
Instruction on how to perform the behaviour	Practical guidance on diet and lifestyle
Information about health consequences	Discussion of the impacts of changes on well-being and health
Cognitive restructuring	Strengthening of self-esteem, identity, and self-confidence
Enhancement of self-efficacy	Promotion of self-care, motivation, and encouragement
Behavioural demonstration	Sharing of experiences during workshops and discussion circles
Social support (emotional)	Support based on Non-Violent Communication (NVC), empathic listening, and strengthening of the professional–patient bond
Reduction of negative stimuli	Modification of harmful eating habits through healthier alternatives
Coping planning	Development of a relapse-prevention plan (Appendix G)
Emotion regulation	Application of mindfulness techniques, emotional regulation, and active listening, including Non-Violent Communication
Knowledge restructuring	Nutrition education adapted to participants' realities
Self-image restructuring	Reflective activities on body image, promotion of body acceptance, and positive self-esteem

Source: Authors' own elaboration, adapted from Michie et al. (2013).

Supplementary Material S3

Checklist for Reporting of Multi-Arm Parallel-Group Randomized Trials: Extension of the CONSORT 2010 Statement

Section/Topic	Item No.	CONSORT 2010 Statement Checklist Item	Multi-Arm Trial Extension	Reported on page no.
**Title and abstract**	1a	Identification as a randomized trial in the title	Identification as a multi-arm randomized trial in the title or an indication of the number of treatment groups that the participants were randomly assigned to	1
	1b	Structured summary of trial design, methods, results, and conclusions (for specific guidance see CONSORT for abstracts)	Specification of the number of treatment groups; details of any groups added or dropped	1
**Introduction**				
Background and objectives	2a	Scientific background and explanation of rationale	Rationale for using a multi-arm design	2-3
	2b	Specific objectives or hypotheses	Specification of the research question referring to all of the treatment groups<br>Clear statement of all hypotheses to be tested and the primary comparisons involved	3
**Methods**				
Trial design	3a	Description of trial design (such as parallel, factorial) including allocation ratio	Specification of the number of treatment groups	4
	3b	Important changes to methods after trial commencement (such as eligibility criteria), with reasons	Details of any treatment groups added or dropped (if relevant), with reasons, and/or changes to the allocation ratio	5
Participants	4a	Eligibility criteria for participants		4
	4b	Settings and locations where the data were collected		4
Interventions	5	The interventions for each group with sufficient details to allow replication, including how and when they were actually administered		5-10
Outcomes	6a	Completely defined prespecified primary and secondary outcome measures, including how and when they were assessed		11-12
	6b	Any changes to trial outcomes after the trial commenced, with reasons		NA
Sample size	7a	How sample size was determined	Planned sample size with details of how it was determined for each primary comparison	12-13
	7b	When applicable, explanation of any interim analyses and stopping guidelines		NA
Randomization				

Sequence generation	8a	Method used to generate the random allocation sequence		4
	8b	Type of randomization; details of any restriction, such as blocking and block size		4
Allocation concealment mechanism	9	Mechanism used to implement the random allocation sequence, such as sequentially numbered containers, describing any steps taken to conceal the sequence until interventions were assigned		4
Implementation	10	Who generated the random allocation sequence, who enrolled participants, and who assigned participants to interventions		4
Blinding	11a	If applicable, who was blinded after assignment to interventions (eg, participants, care providers, individuals assessing outcomes) and how		4
	11b	If relevant, description of the similarity of interventions		NA
Statistical methods	12a	Statistical methods used to compare groups for primary and secondary outcomes	Explicitly state if no adjustments for multiplicity were applied; if adjustments were applied, state the method used	13
	12b	Methods for additional analyses, such as subgroup analyses and adjusted analyses		13
**Results**				
Participant flow (a diagram is strongly recommended)	13a	For each group, the numbers of participants who were randomly assigned, received intended treatment, and were analyzed for the primary outcome		13 – figure 4
	13b	For each group, losses and exclusions after randomization with reasons included		13 – figure 4
Recruitment	14a	Dates defining the periods of recruitment and follow-up	If periods of recruitment and follow-up are different across treatment groups (eg, groups were added or dropped), the periods of recruitment and follow-up, reason(s) for the differences, and any statistical implications should be described	4
	14b	Why the trial ended or was stopped		NA
Baseline data	15	A table showing baseline demographic and clinical characteristics for each group		14 – table 3
Numbers analyzed	16	For each group, number of participants (denominator) included in each analysis and whether the analysis was by original assigned groups		13-14
Outcomes and estimation	17a	For each primary and secondary outcome, results for each group, and the estimated effect size and its precision, such as 95% CI	Results for each prespecified comparison of treatment groups	15-17<br>Tables 4 and 5
	17b	For binary outcomes, presentation of both absolute and relative effect sizes is recommended		12

Ancillary analyses	18	Results of any other analyses performed, including subgroup analyses and adjusted analyses, distinguishing prespecified from exploratory		17
Harms	19	All important harms or unintended effects in each group (for specific guidance, see CONSORT for harms)		18
**Discussion**				
Limitations	20	Trial limitations, addressing sources of potential bias, imprecision, and, if relevant, multiplicity of analyses		20-21
Generalizability	21	Generalizability (external validity, applicability) of the trial findings		21-22
Interpretation	22	Interpretation consistent with results, balancing benefits and harms, and considering other relevant evidence		18-22
**Other information**				
**Registration**	23	Registration number and name of trial registry		1 - abstract
**Protocol**	24	Where the full trial protocol can be accessed, if available		Not available
**Funding**	25	Sources of funding and other support (such as supply of drugs); role of funders		23
